# Investigating the role of “Immature Myeloid Cells” as Drivers of Inflammation and Disease Persistence in Psoriatic Arthritis

**DOI:** 10.31138/mjr.34.2.271

**Published:** 2023-06-30

**Authors:** Alexandros Grivas, Maria Grigoriou, Charalampos Papagoras, Ioannis Mitroulis, Panayotis Verginis, Pelagia Katsimbri, Dimitrios T. Boumpas

**Affiliations:** 1 Biomedical Research Foundation of the Academy of Athens, Greece,; 2 Joint Rheumatology Program, National and Kapodistrian University of Athens, School of Medicine-Clinical Immunology-Rheumatology Unit, 4th Department of Medicine, Athens, Greece,; 3 First Department of Internal Medicine and Laboratory of Molecular Hematology, University Hospital of Alexandroupolis, Democritus University of Thrace, Alexandroupolis, Greece,; 4 Laboratory of Immune Regulation and Tolerance, Division of Basic Sciences, Medical School, University of Crete, Heraklion, Greece

**Keywords:** immature myeloid cells, psoriatic arthritis, response to treatment, blood, skin, synovial fluid

## Abstract

**Background:**

Despite the development of treatments targeting T cell co-stimulation and cytokines TNF, IL-12/23, and IL-17, less than half of patients within clinical trials achieve high levels of clinical response. This fact, as well as the absence of prognostic biomarkers represents major unmet clinical needs that necessitate further investigation of the disease pathophysiology. Myeloid cells are critical components of PsA inflammatory mechanisms, being a highly prevalent immune population in biopsies of PsA target tissues, such as the skin and the synovium. Through their antigen-presenting capacity and their pro-angiogenic and pro-inflammatory properties myeloid cells could contribute to persistent inflammation in PsA leading to treatment-resistant disease. To this end, we have recently shown the expansion of monocytes in the blood of PsA patients compared to healthy subjects. Importantly, we have also identified an immature myeloid cell population in patients with highly active, refractory disease, indicating the presence of an “emergency myelopoiesis” process in PsA.

**Aim of the study:**

In this research protocol, we aim to decipher the pro-inflammatory “myeloid signature” in patients with active PsA and explore the role of immature myeloid cells in disease pathophysiology and their potential as prognostic biomarkers.

**Methods:**

To address this, we will isolate and analyse monocytes and immature myeloid cells from PsA patients -before and after a 6-month treatment course- focusing on differences between responders and non-responders. In this context, we will perform a thorough phenotypic and functional analysis of these cells, identify their expression signature in an already established whole blood RNA-seq dataset and investigate their presence in target tissues, such as the skin and synovial fluid.

**Anticipated benefits:**

This study will elucidate the role of myeloid cells in disease propagation by further defining the involvement of immature myeloid cells in PsA.

## BACKGROUND

Psoriatic Arthritis (PsA) is a chronic immune-mediated disease within the Spondylarthritis spectrum of diseases. It affects up to one-third of patients with psoriasis and manifests with skin and musculoskeletal inflammation.^1,2^ The introduction of biological disease modifying anti-rheumatic drugs (bDMARDS) targeting T cells and cytokines of the TNFα and IL-12/-23 axis has led to a major change in the management of the disease, reflecting the important pathophysiologic role of these cells and of their derived products.^3–5^ Nevertheless, treatment of PsA is still far from ideal, since 30–40% of patients show only a partial response to those treatments and currently there are no prognostic biomarkers guiding the therapeutic approach.^6^ These observations suggest the contribution of additional pathways to disease persistence and implicate the role of other immune cell populations in disease pathophysiology that remain to be identified and further explored.

Myeloid cells, including monocytes, macrophages, neutrophils, and dendritic cells, have been relatively understudied in PsA, but current research points to the importance of this compartment in disease pathophysiology. Mass cytometry and single-cell RNA sequencing-based studies have shown that monocytes are the prevalent cell subset in the synovial fluid of PsA patients, where they contribute to disease perpetuation by secreting inflammatory cytokines (ie, IL8) and chemokines (ie, osteopontin, CCL2, CXCL10).^7^ Monocytes constitute an important source of IL-23 at the entheses of patients with PsA, a tissue with critical importance for the disease’s pathogenesis.^8^ Finally, neutrophils are one of the most abundant cell types in both the skin and joints of patients with PsA.^9^

In chronic inflammatory conditions, such as systemic autoimmune diseases, inflammatory cytokines (IL-1, IL-6, IL-17, IFN-α) and growth factors (G-CSF, GM-CSF) accumulate in peripheral tissues and blood affecting the hematopoietic progenitor cells of the bone marrow by promoting their differentiation into cells of myeloid lineage. This type of “emergency myelopoiesis” leads to the release into the circulation of “immature myeloid cells”, which include Immature Myeloid Cells-IMCs and Myeloid-Derived Suppressors Cells - MDSCs.^10^ The role of these cells has not been adequately investigated in PsA. Studies in experimental models of spondyloarthritis have revealed the process of increased myelopoiesis under the influence of IL-17 and GM-CSF,^11,12^while studies in psoriasis have found an increased number and pathological function of MDSCs.^13,14^ Preliminary experiments in our lab, using flow cytometry, have shown increased frequency of monocytes in the peripheral blood of 30 PsA patients compared to 10 healthy subjects and the presence of immature myeloid cells in the subgroup of patients who do not respond to therapy.^15^

## AIM OF THE STUDY

Our working hypothesis is that PsA patients with highly active disease are characterized by a “myeloid signature” in their blood and target tissues that drives the persistence of inflammation and that this signature could potentially predict response to treatment. To this end, we seek to explore and define the role of myeloid progenitor cells in PsA pathogenesis.

## PATIENTS AND METHODS

30 adult patients with PsA will be recruited in the study and monitored by the Rheumatology clinics of “Attikon” University General Hospital and University General Hospital of Alexandroupolis, after written informed consent, and approval by the Scientific Committees of the two hospitals. Patients will be followed up for a period of six months after the initiation of treatment and will be classified into responders and non-responders according to their response status at the 6-month time point.

Inclusion criteria include: (i) PsA diagnosed based to CASPAR criteria, (ii) Moderate to high activity based on DAPSA (DAPSA >15), (iii) Initiation or change of treatment (conventional synthetic DMARDS csDMARDs, anti-TNF, anti-IL17, anti-IL12/23, PDE inhibitor) (t=0), (iv) Monitor for 6 months +/− 1 month (i.e. a 2-month window) and evaluation of the response to treatment based on ACR50 and/or 75% change of DAPSA (t=6). Additionally, PASI, BSA and LEI score will be assessed during the follow-up. Exclusion criteria include: (i) Age <18 years, (ii) Co-existing autoimmune or autoinflammatory disease or active infection disease, (iii) Pregnancy, (iv) History of malignancy ever.

**Figure 1.**
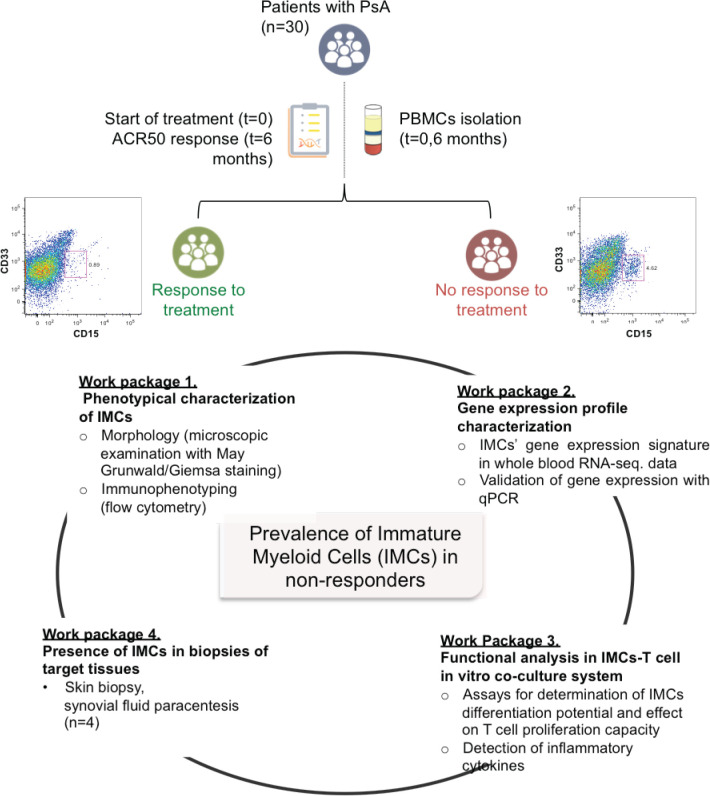
Research methodology summary.

We plan to test our hypothesis through four distinct approaches (**Figure 1**):

### Work package 1

We will isolate immature myeloid cells from peripheral blood mononuclear cells (PBMCs) and perform phenotypic characterisation by flow-cytometry at time point 0 and 6 months. The antibody staining panel that will be utilized is the following: CD3, CD4, CD8, CD19, CD56 for negative selection and HLA-DR, CD14, CD15, CD16, CD33, CD66b for positive selection. Additionally, morphological characterization of isolated PBMCs and immature myeloid cells will be performed by May Grunwald/Giemsa staining.

### Work package 2

We will perform a meta-analysis of published whole blood RNA-seq data from PsA patients to confirm the frequency of immature myeloid cells and to describe their contribution to the whole blood gene expression signature. Results for specific target genes will be validated by qPCR in order to characterize the specific molecular signature of these cells. Monocytes isolated from the same patients will be used as negative control.

### Work package 3

The functional properties of immature myeloid cells will be assessed *in vitro*. Immature myeloid cells and T cells will be isolated from the peripheral blood of patients and will be co-cultured *in vitro*. This experiment will characterise immature myeloid cell’s differentiation potential and their effect on T cell proliferation capacity. The supernatants of the above co-culture system will be examined with LEGENDplex assay that offers the advantage of simultaneous detection of multiple inflammatory cytokines.

### Work package 4

Samples from target-tissues will be examined (n=4/tissue biopsy) for the presence of immature myeloid cells will be performed with a 4-mm punch biopsy, and synovial fluid will be acquired with aspiration from affected joints. Skin biopsies from healthy individuals and synovial fluid from patients with osteoarthritis will be used as negative controls.

## ANTICIPATED BENEFITS

This study will be the first to systematically explore the role of immature myeloid cells as propagators and/or effectors of PsA-related inflammation. The results are expected to reveal new myeloid cell-related biomarkers of response to treatment, and potential novel therapeutic targets.
